# Clinical and Laboratory Profile of Patients Visiting the Post-COVID-19 Clinic at a Tertiary Care Hospital: A Cross-Sectional Study

**DOI:** 10.7759/cureus.22888

**Published:** 2022-03-06

**Authors:** Vijayashree Thyagaraj, Akshay Rao, Ashwin Kulkarni, Tharanath Shankar, Nithin R, Hridya Unnikrishnan, Keerthi Kalaiah, Iniya E, Sundar K Veluswamy, Nanda Kumar B S, Savita Ravindra, Naresh Shetty

**Affiliations:** 1 Internal Medicine, Ramaiah Medical College, Bengaluru, IND; 2 General Medicine, Ramaiah Medical College, Bengaluru, IND; 3 Medicine, Ramaiah Medical College, Bengaluru, IND; 4 Physical Medicine and Rehabilitation, Ramaiah Medical College, Bengaluru, IND; 5 Community Medicine, Ramaiah Medical College, Bengaluru, IND; 6 Orthopaedics, Ramaiah Medical College, Bengaluru, IND

**Keywords:** post-acute covid-19, cough, fatigue, post-covid sequelae, post covid-19 manifestations

## Abstract

Background: Coronavirus disease 2019 (COVID-19) survivors may continue experiencing diverse symptoms. This study portrays the clinical and laboratory profile of patients with post-acute sequelae of COVID-19’(PASC) at a tertiary care hospital in India.

Methodology: This cross-sectional study enrolled patients visiting the post-COVID-19 clinic three weeks after their acute COVID-19 illness. Their clinical, serological, and radiological characteristics were collected and analyzed.

Results: Of the 259 participants (age: 48.02±15.27 years; 62.25% men), 168 had PASC manifestations. The most frequently reported symptoms were fatigue (n=71(42.26%)), breathlessness (n=38(22.61%)), and cough (n=35(20.83%)). Patients with PASC had higher body mass index (28.24±5.02 vs. 26.26±3.65; p=0.002), history of hypertension (52 (30.95%) vs. 17 (18.6%); p=0.039), uncontrolled systolic blood pressure (37 (22.03) vs. 14 (15.38); p=0.042), and persistent chest x-ray abnormalities (34 (20.23) vs. 10 (10.98); p=0.048).

Conclusion: Fatigue, breathlessness, and cough are common PASC symptoms. Hypertension, obesity, and abnormal chest x-ray findings at follow-up are potential risk factors for developing PASC.

## Introduction

The pandemic of coronavirus disease 2019 (COVID-19) has wreaked devastation worldwide. India has reported over 42 million COVID-19 cases as of March 4, 2022 [1]. As the dust from the COVID-19 storm settled, an alarming trend was observed across the world. Several survivors seemed to be experiencing a myriad of protracted symptoms beyond the acute phase of the illness. Terms such as “Long COVID,” “Post-acute COVID-19 illness,” and "Post-acute sequelae of COVID-19 (PASC)" have been used to describe this condition wherein patients continue to have symptoms beyond three weeks from the onset of acute COVID-19 [2,3]. The pathophysiological mechanisms underlying these clinical manifestations are most likely varied among affected patients. The severity of PASC illness also varies from mildly bothersome symptoms to life-threatening emergencies requiring hospitalisation, intensive care, and monitoring. Some people with PASC may also experience a confusing syndrome of relapsing-remitting symptoms [4]. The risk factors predisposing to the development of PASC are yet to be fully ascertained since some patients who remained asymptomatic in the acute COVID-19 phase have gone on to experience PASC symptoms whereas some survivors of even severe disease remained free from symptoms beyond three weeks [5]. 

The symptoms of PASC may include cough, breathlessness, fever, sore throat, chest pain, palpitations, cognitive deficits, myalgia, neurological symptoms, skin rashes, and diarrhoea [6]; some may also have persistently low oxygen saturation [7]. Post-COVID-19 care clinics should ideally be managed by a dedicated team of physicians, pulmonologists, psychologists, cardiologists, and physiotherapists who provide comprehensive treatment and rehabilitation to such patients. There are limited studies from India that describe the clinical manifestations of the patients having PASC and even fewer from the first surge of COVID-19 infections across the country. This study attempts to portray the clinical profile of PASC and the factors associated with it among the patients who visited the post-COVID-19 clinic at a tertiary care hospital. 

## Materials and methods

This is a cross-sectional study carried out at a tertiary care hospital in South India. After obtaining clearance (MSRMC/EC/AP-05/11-2020) from the Institutional Ethical Committee of Ramaiah Medical College, Bengaluru, the study was conducted as per the ethical rules of Declaration of Helsinki. 

In recognition of the rising number of patients seeking medical attention for PASC symptoms, the hospital set up a post-COVID-19 clinic, which became operational on September 7, 2020, and was one of the first such clinics to be established in the country. It is managed by a team of consultants that included specialists from departments of Internal Medicine, Pulmonology, Cardiology, Physiotherapy, Psychiatry, and Neurology. The clinic is equipped with laboratory and radiology services. The hospital had been operating a 500-bedded designated COVID-19 wing from earlier that year where acute COVID-19 infected patients were hospitalized. Patients were advised to visit the post-COVID-19 clinic following their discharge from the hospital. 

The study enrolled all patients who attended the post-COVID-19 clinic from September 7 to December 15, 2020, after obtaining written informed consent. They were included in the study if they had presented at least 21 days after the onset of their acute COVID-19 symptoms. In the case of patients who had asymptomatic acute COVID-19 illness, it was necessary that they had completed at least 21 days after testing positive for COVID-19 (confirmed on reverse transcriptase polymerase chain reaction (RT-PCR) or rapid antigen testing (RAT)), to be included in the study. Patients were excluded if they did not grant consent to participate in the study or if they had presented within 21 days of symptom onset/testing positive for COVID-19. Patients diagnosed to have "COVID-19-like illness" but reported negative on COVID-19 RT-PCR were also excluded. 

The demographic details, comorbidity, and details of the COVID-19 illness were recorded. A pre-designed questionnaire was used to document the various PASC symptoms reported by the participants. This questionnaire included a section on self-reported symptom list that patients were asked to fill out following which they would proceed for their Internal Medicine consultation. The blood pressure (BP) was measured using an Omron HEM automatic BP monitor (Omron Healthcare, Inc., Kyoto, Japan) with the patient seated comfortably for at least five minutes. The pulse rate and peripheral oxygen saturation were recorded using the Omron CMS50N Contec Pulse Oximeter (Omron Healthcare, Inc., Kyoto, Japan). The height in meters (m) and weight in kilograms (Kg) were measured and used to calculate their body mass index (BMI) using the formula - weight in Kg/(Height in m)^2^. As per the clinician’s discretion, the patients were subsequently directed to physiotherapy assessment, other specialist providers as needed, and investigations. All patients underwent investigations like hemoglobin estimation via sodium lauryl sulfate detection, total leucocyte count estimation by direct current detection method, and C-reactive protein (CRP) as well as D-dimer measurement by immune-turbidometry. All patients had a chest radiograph taken in the posterior-anterior view. The chest x-rays taken during acute COVID-19 illness were used for comparison. The x-ray films were interpreted by a team of radiologists. 

In an effort to reduce selection bias, all patients who were being discharged from the acute COVID-19 wing were being advised to follow up at the post-COVID-19 clinic after completion of 21 days from onset of illness/date of testing. Telephonic reminders were sent out prior to their scheduled visit. Advertisements via traditional and social media were put out to spread awareness regarding the services at the clinic and encourage survivors of all categories of acute COVID-19 to visit the clinic. 

Statistical analysis

Collected data was entered into Microsoft Excel sheet (Microsoft Corp., Redmond, Washington), and analysis was carried out using JMP® pro 16 (SAS Institute Inc., Cary, North Carolina). Inferential and descriptive analysis was done. Continuous data were presented as mean +/- SD and categorical data as frequency (%). Normality of distribution for continuous data was determined using the Shapiro-Wilk test. For non-normally-distributed data, the Mann-Whitney U test was employed for the analysis. Student’s t-test was used for normally distributed continuous scale on metric parameters. The Chi-square test was used to compare categorical variables. A p-value of ≤ 0.05 was considered significant. 

## Results

**Figure 1:**
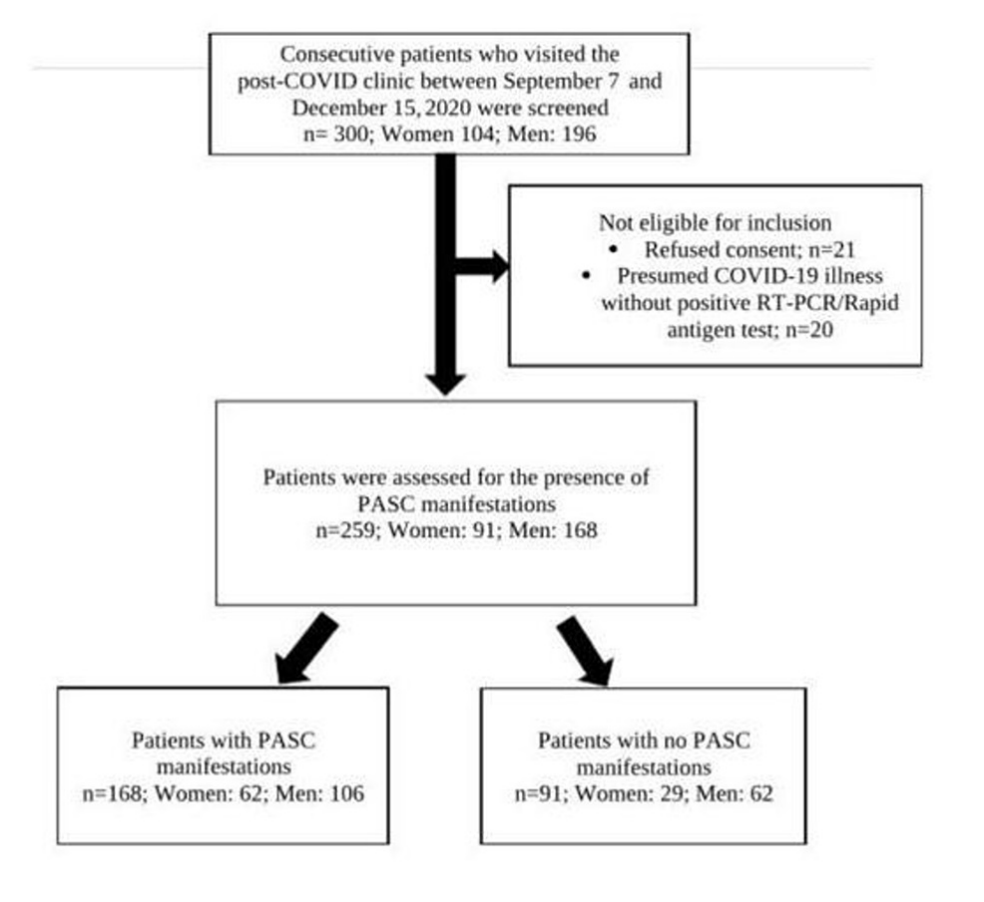
STROBE diagram: recruitment of patients in the study STROBE: strengthening the reporting of observational studies in epidemiology; COVID-19: coronavirus disease 2019, PASC: post-acute sequelae of COVID-19; RT-PCR: reverse transcriptase polymerase chain reaction

The first 300 patients who presented to the post-COVID-19 clinic between September 7 and December 15, 2020, were considered for inclusion in this study. After excluding ineligible patients, the data of 259 patients were used in the final analysis (Figure 1). A significantly high number of male patients (n=168 (65.25%)) sought medical attention at the clinic in comparison to the number of female patients (n=91 (35.13%)). The average age of male patients at the clinic was 49.42 ± 15.19 years whereas for female patients it was 46.63 ± 15.35 years. A large number of patients were in the sixth decade of their life. The majority of patients who presented to the post-COVID-19 clinic were those who had endured a mild COVID-19 illness (40.9%) followed by patients with moderate (32.8%) and severe categories of the disease (20.4%). Fifteen patients who remained asymptomatic after incidentally testing positive on RT-PCR for COVID-19 had also presented to the clinic for routine follow-up. Systemic hypertension was observed in 87 (33.59%) patients and type-2 diabetes mellitus in 69 (26.64%) patients accounted for the most prevalent associated medical conditions in the study group (Table 1). 

**Table 1:** Age, gender, co-morbidities, body mass index, and severity of COVID-19 infection of the study participants COVID-19: coronavirus disease 2019

Parameter	Group	N=259
Gender (n=259)	Female	92 (35.13%)
	Male	167 (65.25%)
Age in years (n=259)	20 – 29	34 (13.12%)
	30 – 39	44 (16.98%)
	40 – 49	50 (19.30%)
	50 – 59	64 (24.71%)
	60 – 69	44 (16.98%)
	70 – 79	19 (7.3%)
	80 - 89	4 (1.5%)
Mean body mass index in Kg/m^2^	Males	26.54±4.23
	Females	28.78±4.60
Category of acute COVID-19 illness	Asymptomatic	15 (5.7%)
	Mild	106 (40.9%)
	Moderate	85 (32.8%)
	Severe	53 (20.4%)
Comorbidities	No comorbidities	109 (42
	Hypertension	87 (33.59%)
	Type 2 Diabetes mellitus	69 (26.64%)
Persistent symptoms beyond 21 days	Yes	168 (64.86%)
	No symptoms	91 (35.13%)

Of the total, 168 (64.8%) patients presented with PASC symptoms while the remaining 91 (35.13%) patients were asymptomatic beyond 21 days from the onset of their acute COVID-19 illness. Fatigue was the most common symptom reported in 71 (42.26%) patients. The next frequent complaints were respiratory complaints like breathlessness seen in 38 (22.61%) patients and cough in 35 (20.83%) patients. Loss of smell was observed in 18 (10.71%) patients, loss of taste in 24 (14.28%), anxiety was seen in 19 (11.3%), and insomnia in 15 (8.92%) patients (Table 2).

**Table 2:** Frequency of symptoms among the study participants PASC: post-acute sequelae of COVID-19 (coronavirus disease 2019)

PASC symptom	Number of patients (%) n=168	Presentation from onset of acute symptoms (days)
Fatigue	71 (42.26)	34.29±16.84
Breathlessness	38 (22.61)	37.91±18.2
Cough	35 (20.83)	35.17±19.68
Loss of taste	24 (14.28)	30.1±7.75
Anxiety	19 (11.3)	36.38±10.97
Loss of smell	18 (10.71)	32.32±9.43
Loose stools	17 (10.11)	23.43±8.42
Insomnia	15 (8.92)	31.33±10.32
Wheezing	7 (4.16)	33.21±10.43
Low mood	7 (4.16)	33.21±10.43

Most of the patients (30.3%; n=51) who presented with post-COVID-19 symptoms visited the hospital 22-28 days (three to four weeks) from the time of onset of acute illness. Three patients in the study had continued to experience symptoms beyond 90 days into their COVID-19 illness (Table 3). Category-wise assessment of patients revealed that 63 (68.47%) patients with moderate COVID-19 illness and 25 (75.7%) patients with severe illness presented with at least one symptom while 73(61.3%) patients with mild acute COVID-19 illness had at least one PASC symptom. However, this difference between the cases was not statistically significant. 

**Table 3:** Comparison of the characteristics of patients who had at least one PASC symptom vs. those who did not SpO2: peripheral oxygen saturation; CRP: C reactive protein; PASC: post-acute sequelae of COVID-19; COVID-19: coronavirus disease 2019

Parameter		Post-COVID-19 symptoms ≥ 1 n=168 (%)	Asymptomatic =91	P-value
Age		49.09 ± 15.19	47.07 ± 15.38	0.312
Gender	Males	106 (63.1%)	62 (75.8%)	0.412
	Females	62 (36.9%)	29 (24.2%)	
Body mass index (Kg/m^2^)		28.24±5.02	26.26±3.65	0.002
Category of illness	Asymptomatic	7 (46.6%)	8 (53.3%)	0.167
	Mild	73 (61.3%)	46 (38.65)	
	Moderate	63 (68.47%)	29 (31.5%)	
	Severe	25 (75.7%)	8 (24.2%)	
Comorbidity	Diabetes	59 (35.12)	28 (30.76)	0.479
	Hypertension	52 (30.95)	17 (18.6)	0.039
	More than one comorbidity	125	43	0.951
Acute COVID-19 symptoms	Fever	89 (52.97)	47(51.64)	0.947
	Cough	63(37.5)	40(43.9)	0.218
	Breathlessness	37(22)	7(7.6)	0.612
	More than one acute symptom	105	63	0.128
Systolic blood pressure > 140 mm Hg		37 (22.03)	14 (15.38)	0.042
Diastolic blood pressure > 90 mm Hg		7 (4.16)	2 (2.19)	0.240
Mean pulse rate/minute				
Mean respiratory rate / minute		18.06 ± 1.03	18.66 ± 0.91	0.143
SpO2 (%)		98.17 ± 0.53	98.13 ± 0.58	0.662
Neutrophil to lymphocyte ratio		2.53 ± 2.27	2.47 ± 1.79	0.414
Haemoglobin (g/dl)		13.93 ± 1.53	13.95 ± 1.50	0.523
Total leucocyte count/ mm^3^		7788 ± 2260	7481 ± 2310	0.322
CRP mg/dl		5.314±8.962	3.712±5.217	0.384
D-Dimer μg/ml		0.692±1.32	0.804±1.44	0.564
Abnormalities on chest x-ray		34 (20.23)	10 (10.98)	0.048

Table 4 summarises the comparison of the characteristics of patients who had at least one PASC symptom vs. those who did not. Among the various parameters to be analysed, a higher BMI (28.24±5.02 vs. 26.26±3.65; p=0.002), a past history of systemic hypertension (52 (30.95%) vs. 17 (18.6%); p=0.039), an elevated systolic BP (37 (22.03%) vs.14 (15.38%); p=0.042) and the presence of persistent abnormalities on chest x-ray (34 (20.23%) vs. 10 (10.98%); p=0.048) were seen to have a significant association with the presence of PASC symptoms. 

**Table 4:** PASC symptoms from time of acute illness PASC: post-acute sequel of COVID-19 (coronavirus disease 2019)

PASC symptoms from time of acute illness	Number of patients n=168
10-21 days	33 (19.64%)
22-28 days	51 (30.3%)
29-35 days	36 (21.4%)
36-42 days	27 (16.0%)
43-49 days	14 (8.3%)
50-90 days	4
Beyond 90 days	3

## Discussion

This cross-sectional study was conducted on patients who presented to a post-COVID-19 clinic at a tertiary care hospital and it offers intriguing insights into the enigmatic phenomenon, PASC. The persistence of symptoms beyond 21 days from the onset of acute COVID-19 illness was noted to be present in 168 (64.86%) out of the 259 patients who presented to the clinic. The most common symptom was fatigue followed by persistent cough and breathlessness. This is in agreement with similar studies conducted by Goërtz et al. in which fatigue and dyspnoea were the most prevalent symptoms during the infection and at follow-up [8]. Similar findings were observed in the study conducted by Carfi et al., where 87.4% reported persistence of at least one symptom, particularly fatigue and dyspnea after the recovery from acute COVID-19 infection [6].

The factors that were seen to be significantly associated with the presence of PASC symptoms in the present study were a higher BMI, past history of hypertension, elevated systolic BP at follow-up, and the persistence of abnormalities on chest radiography. While there have been studies that have shed light on factors that could predict the development of PASC symptoms, certain other studies have failed to report any such findings [9]. The findings of the present study were concordant with the study conducted by Khasawneh et al. in Jordan, which demonstrated that obese patients experienced PASC symptoms almost twice as commonly as patients with a normal BMI [10]. An abnormally high BMI and obesity has been found to be associated with PASC symptoms in other studies. Aminian et al. observed a higher rate of rehospitalization and even delayed mortality during the PASC phase among obese patients. The findings of their study suggested that moderate to severe obesity (BMI ≥ 35 kg/m2) is associated with a greater risk of developing PASC symptoms. Obesity is a state of heightened inflammation owing to the presence of adipokines. The amplification of inflammation during COVID-19 infection has been proposed to predispose obese individuals to more severe illness in acute phase as well as possibly to the development of PASC symptoms [11].

In the present study, both the history of essential hypertension and elevated systolic clinic BP values at follow-up were found to be associated with prevalence of PASC symptoms. Similar findings were observed by Galal et al. who noted that the most frequent pre-existing comorbidities seen with persistent post-COVID-19 symptoms among the study population was hypertension (p= 0.039) [12]. A confounding effect cannot be ruled out since obesity tends to be associated with systemic hypertension [13]. Some authors have proposed that systemic hypertension may be a post-COVID-19 sequela by itself. Angiotensinogen converting enzyme II receptors serve as the binding sites for the COVID-19 virus, which allows the virus to gain entry into the host cells [14]. This leads to an up-regulation of the renin-angiotensin system and is hypothesized as the reason behind the association of systemic hypertension with PASC [15].

Notably, no significant association was apparent between PASC symptoms and gender, age, and severity of the acute COVID-19 illness as has been reported in some earlier studies. A study conducted in China found that women are more likely to experience fatigue at six months follow-up following acute COVID-19 infection [16].

Radiological abnormalities like persistence of ground-glass opacities at follow-up were noted to be associated with at least one PASC symptom in this study. Other studies have reported the persistence of radiographic abnormalities well beyond the acute phase of the illness [17]. Since COVID-19 is primarily a respiratory infection, it can be anticipated that continued pulmonary opacities picked up on thoracic radiography render such individuals susceptible to experience residual respiratory symptoms. The lingering opacities may be indicative of fibrosis that some of the patients with COVID-19 pneumonia go on to develop [18,19]

Hitherto, the mechanisms behind the development of PASC have remained elusive but some potential explanations have been put forward based on previous studies in post-infectious syndromes that occurred following the original severe acute respiratory syndrome viral pneumonia outbreak in China [20]. PASC has been touted to be a form of post-critical illness syndrome and the patients who have suffered from severe acute illness continue to experience lingering symptoms post recovery. Yet, in the current study, there was no significant association between the categories of acute COVID-19 illness and the prevalence of PASC symptoms. Interestingly, in this study, some patients with asymptomatic incidental COVID-19 illness also experienced PASC symptoms, as has been described previously by other researchers. Tabacof et al. reported a range of long-term symptoms in a cohort of previously confirmed or presumed COVID-19 patients whose acute symptoms were largely managed without the need for hospitalization [21]. Findings of Vaes et al. also showed that COVID-19 has a significant impact on care dependency in non-hospitalized patients. Three months after the onset of symptoms, a considerable proportion of patients had persistent complaints and were dependent on others for personal care [22]. PASC has been postulated by some researchers to be due to persistent inflammation beyond the acute phase of the illness [23]. In the present study, there was no indication of persistent systemic inflammation among those with PASC symptoms in comparison to those without. CRP and neutrophil to lymphocyte ratio (NLR), which are considered to be markers of systemic inflammation during acute COVID-19 infection, were not seen to be significantly elevated in these patients. The levels of D-dimer also did not significantly differ between the two groups. Yet another theory advocates that PASC is a result of virus-induced pathophysiological changes taking place within various organ systems [24]. Large-scale studies are warranted in the future to uncover the underlying mechanisms responsible for PASC.

The management of PASC requires a holistic approach and comprehensive care. Perego et al. have suggested that illness due to PASC should have a fair recognition on a global scale and have called for care, equity, compassion, and collective action - involving prominent stakeholders. They have advised a comprehensive patient-focused approach incorporating workup and management with empathy [2]. A similar approach was suggested by Greenhalgh et al., who said that many patients with prolonged COVID-19 illness need a holistic and paced approach through inter-professional, community rehabilitation services [3]. Ladds et al. have suggested quality principles for a long COVID-19 service include ensuring access to care, multi-disciplinary rehabilitation, evidence-based investigation and management, and further development of the knowledge base and clinical services [4]. Houben-Wilke et al. have also suggested follow-up and care after COVID-19 illness, including physiotherapy and exercises at the right time [24].

The limitations of this study are its relatively small sample size and its cross-sectional design. The advantage of the study is that it was carried out in a period before the COVID-19 vaccination began, hence providing valuable insights into the PASC produced during the first wave of COVID in the country as well as offering a comparison for post-vaccination PASC studies. Further large-scale prospective randomised trials are required to uncover more information about PASC.

## Conclusions

PASC manifestations can be quite myriad and warrant extended follow-up and continuing care. In this single-center observational study conducted at a dedicated post-COVID-19 clinic during the first wave of cases in this country, it was found that a significant proportion of patients who had been infected with COVID-19 had persistent symptoms beyond 21 days of the onset of their illness. Among these, fatigue was the most common complaint followed by cough and breathlessness. Those with higher BMI and systemic hypertension were seen to be more frequently associated with PASC manifestations. Neither the gender nor the category of acute COVID-19 illness severity were associated with the development of PASC symptoms in this study. In view of the ever evolving nature of the virus leading to to emergence of several variants, this study provides useful insights and these findings can be compared with PASC manifestations observed in the subsequent waves of COVID-19.
